# Bioprinting and Preliminary Testing of Highly Reproducible Novel Bioink for Potential Skin Regeneration

**DOI:** 10.3390/pharmaceutics12060550

**Published:** 2020-06-13

**Authors:** Forough Hafezi, Susan Shorter, Atabak Ghanizadeh Tabriz, Andrew Hurt, Victoria Elmes, Joshua Boateng, Dennis Douroumis

**Affiliations:** 1 School of Science, Faculty of Engineering and Science, University of Greenwich, Chatham Maritime, Kent ME4 4TB, UK; f.hafezi@greenwich.ac.uk (F.H.); susan.shorter@greenwich.ac.uk (S.S.); a.ghanizadehtabriz@greenwich.ac.uk (A.G.T.); a.hurt@greenwich.ac.uk (A.H.); v.elmes@greenwich.ac.uk (V.E.); 2 Centre for Innovation and Process Engineering Research, University of Greenwich, Chatham Maritime, Kent ME4 4TB, UK

**Keywords:** 3D bioprinting, bioink, chitosan, genipin, human dermal fibroblasts, primary epidermal keratinocytes, skin regeneration

## Abstract

Three-dimensional (3D) bioprinting is considered as a novel approach in biofabricating cell-laden constructs that could potentially be used to promote skin regeneration following injury. In this study, a novel crosslinked chitosan (CH)–genipin (GE) bioink laden with keratinocyte and human dermal fibroblast cells was developed and printed successfully using an extruder-based bioprinter. By altering the composition and degree of CH–GE crosslinking, bioink printability was further assessed and compared with a commercial bioink. Rheological analysis showed that the viscosity of the optimised bioink was in a suitable range that facilitated reproducible and reliable printing by applying low pressures ranging from 20–40 kPa. The application of low printing pressures proved vital for viability of cells loaded within the bioinks. Further characterisation using MTT assay showed that cells were still viable within the printed construct at 93% despite the crosslinking, processing and after subjecting to physiological conditions for seven days. The morphological study of the printed cells showed that they were mobile within the bioink. Furthermore, the multi-layered 3D printed constructs demonstrated excellent self-supportive structures in a consistent manner.

## 1. Introduction

Additive manufacturing (AM) also referred to as 3D printing [1], enables layer-by-layer fabrication to generate 3D objects by employing computer aided design (CAD). There are various AM techniques available for fabricating 3D constructs to aid in skin regeneration. These include selective laser sintering (SLS), which is very versatile and can precisely pattern relevant cells to fabricate 3D tissues constructs [2]; inkjet printing [3], which is used to produce consecutive layers of hydrogels and cells on top of each other to methodically achieve 3D cellular assemblies containing cells. Extrusion/deposition [4] -based techniques are employed for extrusion printing of highly viscous cell-laden hydrogels and can allow easy flow of such hydrogels through the nozzle without the need to apply high temperatures. Bioprinting is a technique for fabrication of 3D objects by deposition of bioinks where cell-laden fluid materials containing additional matrix components are loaded into 3D printers for fabricating various constructs in a layer-by-layer manner. It combines AM, biology, and material science, enabling the construction of 2D and 3D biostructures by incorporating cells and other biologically active entities in predefined locations [5,6,7,8,9]. The native organs and tissues, such as skin, are complex structures consisting of different cell types and extracellular matrix (ECM) materials. Traditional tissue engineering strategies have not been able to fully reproduce biomimetic and heterogeneous tissue constructs because of the lack of appropriate biomaterials and technologies. However, recently developed 3D bioprinting techniques can be leveraged to produce biomimetic and complex tissue structures. To achieve this, multicomponent bioinks composed of multiple biomaterials, different types of cells, and soluble factors have been developed [10,11,12].

As a result, bioprinting has gained increased attention for advanced fabrication and bio-engineered tissue construction [13]. However, 3D bioprinting techniques for the industrial production of customised 3D functional living constructs for regeneration and restoration of tissue and organ function face significant ethical barriers. These include social stratification of bioprinting, managing expectations of untested paradigms (living cells), and ownership of printed bio-objects. It is therefore critical to explore and identify these ethical issues before therapeutic 3D bioprinting is ready to be widely used in human patients [14,15].

Current 3D bioprinting research has generated promising outputs towards the production of skin substitutes for patients who have suffered extensive skin damage due to severe burns, chronic ulcers, cancer and surgery [16,17]. The first generation of engineered skin was made possible through conventional tissue engineering using biodegradable scaffolds by seeding cells into the scaffold matrix and addition of growth factors into these matrices, to assist cell growth and proliferation. However, such strategies can be time consuming and more importantly, there is not much control over the structures’ geometry [18,19]. Though skin substitutes facilitate better skin regeneration than commonly used wound dressings, they are hampered due to technological limitations in fabricating multi-layered structures which mimic the skin, where each layer comprises cells and appendages, including their mechanical fragility and increased costs [20]. Three-dimensional printing approaches have been successfully used for bioprinting of skin by employing inkjet, bio-extrusion, and laser-based techniques [21,22].

Different biomaterials, such as natural polymers (collagen, gelatine), have been applied to prepare bioinks for fabricating various tissue constructs [10]. However, these materials generally suffer from low mechanical properties, and therefore, there is a need for other biomaterials to be employed to obtain bioinks with improved properties [23]. Such ideal properties include rheological (viscoelastic) characteristics of bioinks to aid printability and obtain structures that maintain their structural and mechanical integrity during multi-layer bioprinting, handling and eventual application [10,14]. Furthermore, such multicomponent bioinks should be biocompatible and biodegradable.

Chitosan (CH) is a biocompatible and biodegradable polysaccharide derived from chitin [24] and CH-based scaffolds have active pharmacological action on various wound healing phases including haemostasis and antibacterial activity [25,26,27]. Genipin (GE) is a crystalline chemical compound and is extracted from gardenia fruits [28]. GE has generally replaced crosslinkers such as glutaraldehyde due to advantages such as exhibiting pharmacological activity (e.g., anti-inflammatory and antibacterial effects) which together with biocompatibility make GE relevant for skin regeneration and wound healing applications [29]. Scaffolds of crosslinked CH with GE have been reported to show good biocompatibility [30], but are mainly known for enhancing the mechanical properties of 3D printed scaffolds. CH–GE and chitosan–polyethylene glycol (CH-PEG) bioinks have been studied in 3D printing/bioprinting applications [10,31,32,33,34]. There are pros and cons to using these hydrogels and researchers attempted to modify these polymers to improve their properties such as bioactivity, mechanical strength, and printability [35]. CH as a bioink on its own has slow gelation rate and poor mechanical properties without modification, but crosslinking of CH gives it good viscoelasticity, since one of the key factors in printability is considered to be associated crosslinking mechanisms [36]. Sodium alginate is a low cost, biocompatible and biodegradable polymer derived from brown algae that has been widely used for hydrogel formation [37]. It is probably the most widely used biomaterial in 3D bioprinting because of its biocompatibility, and reversible control over stiffness [38]. Sodium alginate also exhibits a fast gelation rate, where it forms hydrogels through ionic crosslinking arising from exchange between calcium and sodium ions [39]. It was therefore employed to enhance the rigidity of the produced constructs to maintain structural integrity.

The choice of appropriate cells is one of the factors that plays an important role in the success of tissue engineering [19]. The most common type of cells applied extensively in skin bioprinting are keratinocytes (KC), which form the outer epidermal layer, and human dermal fibroblasts (HDF), which are present in the dermal layer of natural skin. There are many advantages in using KCs in skin tissue regeneration after injury [40]. HDFs are used in dermal skin substitutes and have the ability to differentiate from highly proliferative progenitor fibroblasts. They are static in nature and provide support and maintenance to the tissues. The inherent problem of long-term fragility of KCs can be overcome by having a layer of dermal fibroblasts underneath its layer [41].

Epidermal–dermal interactions are known to regulate keratinocyte proliferation and co-culturing of both cells has been reported in the literature as contributing to the effective regeneration of skin wounds. For example, Shephard and co-workers [42] investigated how HDFs respond to epithelial stimuli by characterising them in mono-layer co-culture with KCs and showed that myofibroblast differentiation was induced in the KC–HDF co-cultures. In addition, Werner and co-workers [43] also stated in their comprehensive review that “KCs stimulate HDFs to synthesise growth factors, which in turn will stimulate KC proliferation in a double paracrine manner”.

Herein, we have developed a printable form of CH hydrogel crosslinked with GE, which was further optimised to print cell-laden bioinks comprising HDFs and KCs. Currently, most of the skin printing studies apply KC cells through post-printing processes such as manual seeding (to seed KCs into a 3D printed scaffold to fix and/or regenerate damaged tissues, which involves human intervention, and therefore, it is not fully automated) [22]. In the case of droplet printing/laser printing (placing cells into special patterns with the help of laser light), cell patterning is limited to 2D patterning and it is difficult to fabricate 3D tissues constructs [19,44]. In this study, we printed for the first time KC and HDF cells embedded in CH–GE-based inks by applying an extrusion bioprinting technique without damaging the cells.

## 2. Materials and Methods

### 2.1. Materials

Low molecular weight (MW) CH (with ≥75% deacetylation), PEG (MW 600), MTT reagent, Triton-X-100, DMSO, PBS (pH 7.4 ± 0.1) and sodium alginate were all purchased from Sigma-Aldrich (Gillingham, UK). GE (98%) was obtained from Linchuan Zhixin Biotechnology (Linchuan, China). Acetic acid, sodium chloride, 4′,6-diamidino-2-phenylindole, dihydrochloride (DAPI) (blue fluorescence), calcein (green fluorescence) and trypan blue were purchased from Fisher Scientific (Loughborough, UK). PBS (pH 7.4 ± 0.1) and MTT reagent were sterile filtered before use. Fetal bovine serum and penicillin/streptomycin were obtained from Gibco (Paisley, UK). HDF cells (ATCC^®^ SCRC1041™), KC cells (ATCC^®^ PCS200011™), DMEM (ATCC^®^ 30-2002^™^), dermal cell basal medium (ATCC^®^ PCS200030 ™) supplemented with KC growth kit (ATCC^®^ PCS200040 ™), and trypsin (ATCC^®^ PCS-999-003™) were obtained from ATCC (Manassas, VA, USA).

### 2.2. Culturing of Primary Human Epidermal Keratinocytes (KC) and Human Dermal Fibroblasts (HDF) Cell Lines

The KC and HDF cells were cultured according the ATCC protocol. KCs were cultured in dermal cell basal medium (37 °C in 5% CO_2_) supplemented with KC growth kit. HDFs were cultured in DMEM supplemented with 10% FBS and 1% penicillin/streptomycin at the same temperature and CO_2_ concentration as KCs. Both KCs and HDFs were incubated in an incubator with medium changes every 48 h and sub-cultured when cells reached 80–90% confluence. Trypsin-EDTA was used to detach the cells by following ATCC protocol, passaged twice a week in tissue culture flasks and were discarded after 6 passages to make sure that the key characteristics of the cells were maintained.

### 2.3. Rheological Characterisation

The rheological properties of CH–GE–PEG (1.2% *w/v* CH, 1.2% *w/v* PEG) hydrogels having different concentrations of GE (0.5–2% *w/v*) were evaluated at 37 °C and room temperature (RT) and compared to commercial bioink (CELLINK START^®^, CELLINK Gothenburg, Sweden). The viscosity profile measurement of each bioink was carried out using an Anton Paar rheometer (Anton Paar, Graz, Austria). Samples were placed between 25 mm diameter circular plates and rheological experiments were performed at shear rates ranging from 0.1 to 100 s^−1^ and the corresponding viscosity was obtained. CH–GE–PEG hydrogels were mixed with DMEM (representative of cells) at 5:1 ratio of hydrogel to medium because at this ratio, the viscosity was not affected. All experiments were performed in triplicate.

### 2.4. ^1^H-^13^C CP NMR Spectroscopy

^1^H-^13^C cross polarisation (CP) MAS NMR spectra of CH, GE and the printed CH–GE film were recorded on a JEOL JNM-ECP 300 MHz spectrometer. Samples were prepared in ZrO rotors with a spin speed of 4.5 kHz. ^1^H-^13^C CP MAS NMR spectra were acquired with a relaxation delay of 4 s, a contact time of 1 ms, an acquisition time of 0.21 s and up to 10,000 scans. Chemical shifts were quoted in ppm from tetramethylsilane (TMS).

### 2.5. Bioink Preparation and Bioprinting of Cell-Laden CH–GE–PEG Hydrogels

The experimentally designed bioinks were prepared as described below:

(a) Alginate bioink.

Sodium alginate 6% *w/v* was cross linked (30 s) with CaCl_2_ (1.2% *w/v*, 0.1 mol/L) in deionised water (DIW) at room temperature (RT). The CaCl_2_ was filtered to remove undissolved solids before use and mixed with sodium alginate (6% *w/v*) at a volume ratio of 1:1, which resulted in a hydrogel which was partially crosslinked. The resulting hydrogel was then autoclaved under standard liquid sterilisation conditions. To confirm that the autoclaving did not significantly change the behaviour of the bioinks, the rheological properties of bioinks were measured and compared with the corresponding non-sterile bioinks.

(b) Chitosan crosslinked cell-laden bioink.

CH bioinks at a concentration of 1.2% *w/v* were prepared by dissolving CH powder and PEG (1.2% *w/v*) in acetic acid (0.25% *v/v*, 0.041 mol/L) solution prepared in DIW at RT. 5 mL of GE (1% *w/v*) was added into the CH solution for 30 min with continuous stirring, which formed an irreversible hydrogel. The resulting hydrogel solution was then autoclaved under standard liquid sterilisation conditions. HDFs and KCs were washed in 1× PBS, trypsinised (5 min) and centrifuged (1100 rpm) for 5 min. The resulting cell pellets were re-dispersed in DMEM and dermal basal medium, respectively, stained with trypan blue and counted with a haemocytometer. After counting, both KCs and HDFs were mixed and centrifuged at 1100 rpm for 5 min and the resulting cell pellet was resuspended in 1 mL DMEM. DMEM was chosen as the medium for subsequent culture with the bioprinted constructs because in preliminary studies (data not shown), the samples dissolved in the dermal cell basal medium.

Subsequently, 2.4 mL of the previously prepared CH–GE–PEG bioink was transferred into 3 mL BD Luer-Lok™ sterile syringe (Becton Dickinson, Franklin Lakes, NJ, USA) and 0.2 mL mixture of KCs and HDFs suspension was transferred into 1 mL BD Luer-Lok™ sterile syringe (Becton Dickinson, NJ, USA). A 5:1 ratio of CH–GE–PEG hydrogel to KC–HDF cells resulted in a uniform cell suspension without affecting the viscosity. In total, 0.5 × 10^6^ cells/mL HDFs and 0.5 × 10^6^ cells/mL KCs were used in the preparation of the cell-loaded CH–GE–PEG bioink. The mixing of the cells and the CH–GE–PEG hydrogel was achieved with the help of a Luer Lock Adapter (CELLINK), which was used to connect the two syringes together and avoid any waste of the material. Both cell-laden bioinks and alginate crosslinked bioinks were transferred into sterile 3 mL printing cartridges (37 °C) individually.

### 2.6. 3D Bioprinting Set Up

**Figure 1:**
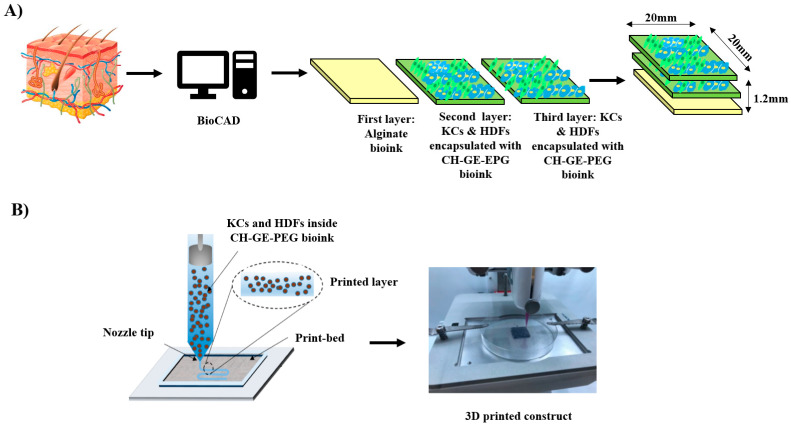
(**A**) Schematic illustration of the design of each layer of skin 3D printed constructs. (**B**) 3D bioprinting of alginate layer and KCs and HDFs encapsulated with CH–GE–PEG layers.

BIO X (CELLINK AB) was used for the printing of CH–GE–PEG and alginate hydrogels. The CH–GE–PEG and alginate inks were loaded into two separate sterile printing cartridges and the printing process was conducted using extrusion-based print-heads. The material flow for each print-head was controlled by individual pressure and speed regulators. Predefined structures were implemented using BioCAD (BIO X software, CELLINK) (Figure 1A). The printability of CH–GE–PEG prepared using three different GE solutions (0.5, 1 and 2% *w/v* GE), using a combination of different printing pressures, speed rates and infill densities (20–40 kPa pressure, 4–6 mm/s speed, 30–50 infill density) and alginate hydrogels (5–12 kPa pressure, 5–7 mm/s speed, 25–40 infill density) was evaluated (Table 1) using a sterile high precision smooth flow tapered nozzle (Nordson-Asymtek, Carlsbad, CA, USA) with 22 G needle (0.41 mm inner diameter) for CH–GE–PEG and 20 G needle (0.58 mm inner diameter) for alginate, and was compared to the commercial bioink (CELLINK START^®^, CELLINK). Hydrogels were printed subsequently to form three layers with a rectangular design (20 × 20 × 1.2 mm, total thickness of 1.2 mm) (Figure 1A).

### 2.7. 3D Printing of Bioinks Constructs

The viability of the cell suspension in culture media was evaluated using a trypan blue exclusion test before bioprinting. The viability was determined as the ratio of number of trypan blue negative cells to the total number of cells. Crosslinked alginate bioink and CH–GE–PEG cell-laden bioink were both printed using a 20 G nozzle (CELLINK BIOX bioprinter (CELLINK)). All bioink components were sterilised prior to printing by using the UV assembly of the printer and the print-head was heated at 37 °C. Rectangular 3D constructs containing alginate as the first layer were printed and exposed to CaCl_2_ (5% *w/v*, 0.450 mol/L) (30 s) to fully crosslink the alginate bioink. Subsequently, cell-laden CH–GE–PEG bioinks (0.5 × 10^6^ cells/mL HDFs and 0.5 × 10^6^ cells/mL) as the second and third layer (20 mm long × 20 mm wide × 1 mm thick) were printed. The bioprinted constructs were then incubated (37 °C, 5% CO_2_) for 7 days, submerged in growth medium (DMEM) and evaluated for viability of the KC and HDF cells over 7 days.

### 2.8. Scanning Electron Microscopy (SEM)

The visualisation of morphology of the cells and their distribution within the 3D bioprinted constructs were undertaken using SEM (SU 8030, Hitachi High Technologies, Krefeld, Germany). The media were aspirated and the 3D bioprinted constructs were then transferred to 6 well plates and fixed by adding 1 mL of 2.5% *w/v* glutaraldehyde (Agar Scientific, Stansted, UK) in PBS and stored for 1 h at RT in a fume cupboard. After 1 h, the glutaraldehyde was aspirated, and the structures were washed with 2 mL PBS twice for 20 min per wash. After removing PBS completely from the wells, 2 mL of 1% *w/v* osmium tetroxide (Agar Scientific, Stansted, UK) was added into each well and incubated at RT for 1 h. Structures were then washed with PBS and initially dried using increasing concentrations of graded ethanol in PBS over a concentration range from 30–100% with 10% increments. Each concentration was applied and allowed to equilibrate for 20 min at RT. The process was repeated twice and dried completely by adding 2 mL of hexamethyldisilane (HMDS) (Agar Scientific, Stansted, UK) to each bioprinted construct and stored at RT in a fume cupboard for 20 min. HMDS was then removed from the wells and the structures were left to dry overnight. Once dried, samples were coated with chromium before SEM analysis. SEM images were captured using accelerating voltage of 1 kV at different magnifications using i-*scan* 2000 software.

### 2.9. Cell Viability of Bioprinted Constructs

To determine the viability of HDFs and KCs within the CH–GE–PEG bioink and the commercial bioink, MTT assay was performed. CH–GE–PEG cell-laden bioinks were bioprinted inside 96 well microtiter plates using a BIO X bioprinter at concentrations of 0.5 × 10^4^ cells/mL KCs and 0.5 × 10^4^ cells/mL HDFs. The bioprinter deposited 100 μL (6.4 mm width × 3 mm thickness) of the bioink inside each well and 100 μL of DMEM was added on top of the bioink in each well. The 96 well microtiter plate was then incubated (37 °C, 5% CO_2_) overnight. After that, MTT reagent (10 μL) was added to the wells and incubated for 4 h. Consequently, the supernatant was aspirated and DMSO (100 μL) was added to dissolve the blue formazan crystals obtained from MTT due to the succinate dehydrogenase within the mitochondria of the cells [26]. The absorbance was measured at 492 nm using a microplate reader and the assay was performed over 7 days. It is worth mentioning that all 7 samples were bioprinted at once and kept in the incubator, and each day, one of the samples was prepared for MTT assay. The average of three (*n =* 3) technical replicates of CH–GE–PEG (that is, repeated measurements of the same sample that show independent measures of the variability associated with the equipment and the protocols) was calculated for each sample tested and percentage cell viability computed with the help of Equation (1):

(1) $$\mathrm{Cell}\;\mathrm{viability}\;\left(\%\right)=\frac{\mathrm{At}\;-\;\mathrm{Ab}}{\mathrm{Ac}\;-\;\mathrm{Ab}}\times 100$$

where At, Ab and Ac are the absorbance of tested samples, medium only and untreated cells, respectively. Untreated cells and Triton-X-100 (0.01% *w/v*) treated cells were used as negative and positive controls, respectively. To investigate the impact of the bioprinting as well as toxicity of hydrogel, similar experiments were conducted with a 7-day culture period. The medium was replaced every 72 h until the 7th day.

### 2.10. Viability Testing of Dispensed Cells by Live/Dead Staining

The viability of the cells within the bioprinted 3D constructs was further investigated using a live/dead fluorescence assay on days 0, 3, 5 and 7 (0.5 × 10^6^ cells/mL HDFs and 0.5 × 10^6^ cells/mL). Calcein (Invitrogen, green fluorescence) was used as a marker of viable cells and DAPI (Fisher Scientific, Waltham, MA, USA, blue fluorescence) was used as a marker to indicate total number of cells. Prior to staining, PBS was used to rinse each sample, after which, they were stained and incubated for 30 min, and then, washed twice with 1× PBS. A confocal microscope (Zeiss LSM 880) was used to acquire the images by averaging each image 4 times. Images were acquired using Zen Black, with a 10× objective, using two tracks, set up for DAPI and calcein. Z stacks were captured, resulting in images of CH–GE–PEG cell-laden constructs with a height of 400 μm and a length of 800 μm. After counting 3 different fields, cell viability was calculated using Equation (2). The reconstructed images were quantitatively analysed with the help of Image J 1.48 v software [45] and used to score the number of green/blue fluorescent cells embedded per unit volume of CH–GE–PEG cell-laden bioink [46].

Cell viability = (number of green stained cells/number of total cells) × 100%(2)

### 2.11. Statistical Analysis

Statistical analysis was carried out to compare MTT and rheological analysis results of bioinks using one-way ANOVA. The results were expressed as mean ± standard deviation and significant differences were measured at a level *p* < 0.05.

## 3. Results and Discussion

### 3.1. Rheological Analysis

Rheological properties play an important role in determining the printability of hydrogels, where various crosslinker concentrations can affect the rheological behaviour, which will directly impact the printability in extrusion bioprinting [14,47]. The hydrogel should be viscous enough to enable dispensing of free-standing strands with suitable strength while providing sufficient stability for the construct [36].

Normally in extrusion 3D bioprinting, an increase in viscosity is correlated with an increase in printing fidelity bioinks, which possess higher viscosities, and appropriate gel strength, which would help to maintain the shape of the printed filament after deposition [36]. However, excessively high viscosity arising from high hydrogel concentrations in the bioinks impedes mass transport, cell proliferation and cell migration, and subsequent deposition of ECM [48]. This therefore requires a balance between high viscosity and appropriately flexible bioinks prior to printing. Furthermore, as the crosslinking agent significantly affects the viscosity, and consequently, the printability, bioinks with different concentrations of GE (0.5–2% *w/v*) were prepared, evaluated and compared to a commercial bioink in order to determine a suitable GE concentration at suitable viscosity range to allow effective printing.

**Figure 2:**
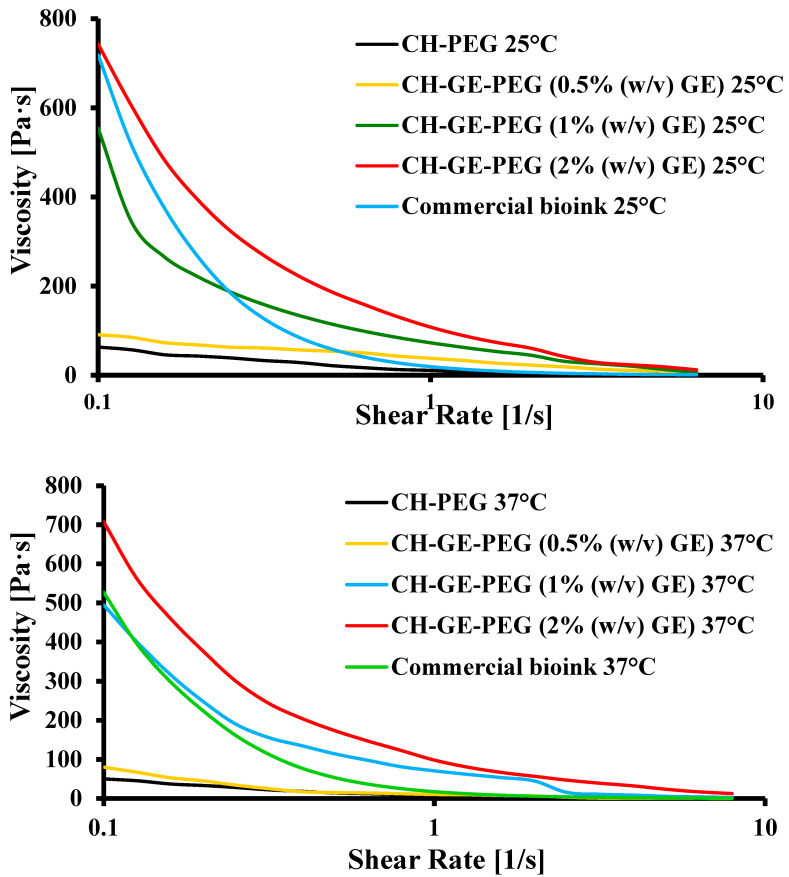
Rheological characterisation of hydrogels; CH–PEG, CH–GE–PEG having different GE% (0.5, 1, 2) and commercial bioink at 25 °C and 37 °C.

The viscosity of CH–PEG and CH–GE–PEG (0.5 (yellow), 1 (blue) and 2 (red) % *w/v* GE) formulations at 37 °C was nearly six-fold lower than the viscosity at 25 °C, which revealed a significant effect of temperature on the bioink viscosity (Figure 2). The viscosity of all bioinks was optimised at 37 °C to facilitate effective cell printing. As shown in Figure 2, increasing shear rates demonstrated a decrease in viscosity of the bioinks, which shows a shear thinning behaviour with increasing pressure, denoting non-Newtonian behaviour. Table 2 shows the rheological properties of crosslinked CH–GE bioinks at various GE concentrations and commercial bioink. The CELLINK START^®^, which is water soluble, was as a representative material for cell-laden constructs. CELLINK START^®^ readily dissolves after washing with culture media and prints at RT using the MCR 702 twin drive rheometer (Anton Parr, Graz, Austria) equipped with metal parallel plate geometry (Anton Paar PP50, 50 mm in diameter).

Viscosity measurements of the CH–GE–PEG in comparison to the CH–PEG blends showed that the addition of GE enhanced the viscosity considerably (Table 2). At the shear rate of 1 s^−1^ over time, the viscosity of the two bioinks decreased slightly (at both 25 and 37 °C). In the case of CH–GE–PEG (1% *w/v* GE), viscosity decreased from about 554.2 and 494.8 to 73 and 70.8 Pa s, respectively, while CH–PEG decreased from 63.4 and 50.3 to 50.3 and 9.1 Pa s, respectively. It was observed that for CH–PEG and CH–GE–PEG (0.5% *w/v* GE) bioinks, the structures were unstable, and collapsed during printing. The CH–GE–PEG (1% *w/v* GE) and commercial bioinks were successfully printed while the CH–GE–PEG (2% *w/v* GE) bioink required higher pressures due to its higher viscosity. For this reason, CH–GE–PEG (1% *w/v* GE) was identified as the most suitable bioink for the purposes of the study, as its rheological characteristics were similar to the commercial bioink. Significant difference (*p* < 0.05) was found between the mean viscosity of CH–PEG and CH–GE–PEG prepared with different concentrations of GE (0.5%, 1% and 2% *w/v*), which could be attributed to the addition of GE.

### 3.2. ^13^C-NMR Spectroscopy

**Figure 3:**
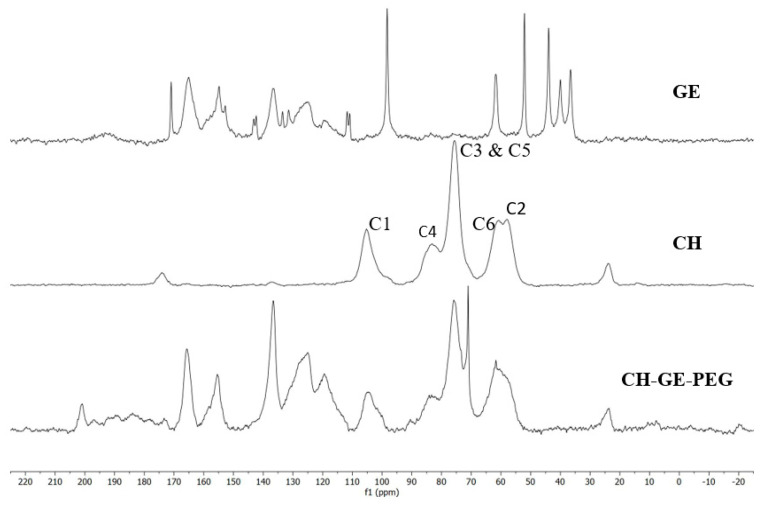
^1^H-^13^C-NMR spectra of CH, GE and the CH–GE–PEG 3D printed film.

Figure 3 shows the ^1^H-^13^C CP MAS NMR spectra of CH, GE and the film (20 × 20 × 0.3 mm) synthesised using the CH and PEG, with GE as a crosslinker. By comparison, the results produced are similar to those observed in the literature [49]. The sharp peaks at 61.6 and 71 ppm are thought to come from the starting material PEG [50]. The reduced visibility of the C4 peak in the film spectrum is due to the crosslinking between CH/PEG and GE [49]. The C6 peak of CH also disappears during the crosslinking process and is typically seen as a shoulder peak to C2, but this is difficult to observe due to the overlap of the PEG peak at 61.6 ppm. The chitosan peak C1 should have a frequency shift when crosslinked with GE (from 105 ppm to 100 ppm), however, this has not been observed in the film ^1^H-^13^C CP MAS NMR spectrum. This could also be due to the presence of PEG in the film. The following NMR data support the other characterisation methods used to demonstrate the crosslinking of the CH and PEG film with GE.

### 3.3. 3D printing of Cell-Laden Constructs Using CH–GE–PEG and Alginate

The schematic diagram in Figure 1 summarises the printing steps followed to obtain cell-laden 3D constructs. Optimisation was performed for various printing parameters including pressure, dispensing speed, infill densities, printing temperature as well layer height. The temperature of the syringe barrel was set to 37 °C and the bioink was smoothly extruded while applying pressure. Seidal and co-authors bioprinted their cell-laden bioinks, applying extrusion-based 3D bioprinting [51]. Their dispensing of the bioink was done through a needle with an inner diameter of 610 μm, at a speed of 8–10 mms^−1^ and a pressure of 80–100 kPa. However, the bioprinted constructs in this study were bioprinted by applying low pressure (20–40 kPa) with a smaller nozzle (400 μm), which reduces the sheer stress exerted upon the cells at the tip of the nozzle. This was important to prevent possible loss in cell function and viability as well as potential clogging of the nozzle [52]. It is essential to ensure high levels of control (print-head temperature, printing speed and pressure) during the deposition of bioinks, when fabricating self-supporting constructs loaded with cells. Precise control of scaffold thickness is achieved by regulating the thickness of one layer (three layers of 400 μm each, which makes the 3D bioprinted construct have a thickness of 1.2 mm) or printing different layers.

**Figure 4:**
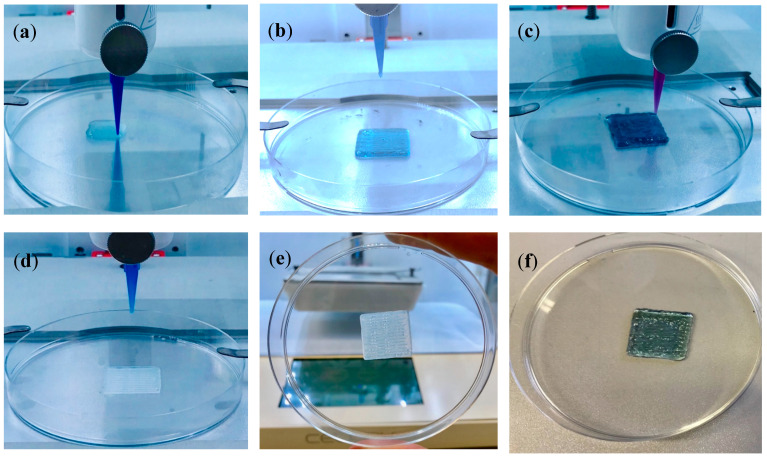
(**a**) CH–GE–PEG 3D printed constructs having 0.5% *w/v* GE, (**b**) CH–GE–PEG 3D printed constructs having 1% *w/v* GE, (**c**) CH–GE–PEG 3D printed constructs having 2% *w/v* GE, (**d**) bioprinting of alginate as first layer, (**e**) bioprinted alginate layer from another view, (**f**) bioprinted alginate/CH–GE–PEG/KCs–HDFs 3D constructs with 1% GE.

In the preliminary stages of the current study, the 3D printing process was optimised to obtain CH hydrogels with precise and controlled structure prior to incorporation with cells. A CH–PEG solution was first prepared and used to produce scaffolds with a mesh structure having different infill densities. An ideal printable material should incorporate a cyto-compatible crosslinker with sufficiently high viscosity to ensure good shape fidelity as well as good material stability under physiological conditions. Although a highly viscous hydrogel would result in a better shape fidelity, it is a prerequisite not to apply high dispensing pressure as it would induce a significant decrease in cell viability [53]. It is therefore important to control the degree of crosslinking within the biomaterials to obtain a suitable printing viscosity. Based on our previous study [26], a blend of 1.2% CH at 1:1 CH: PEG ratio was selected for the preparation of the bioink, while different concentrations of GE were evaluated (0.5, 1 and 2% *w/v*) to obtain an appropriate viscosity. As shown in Figure 4a, CH–GE–PEG 3D printed constructs with 0.5% *w/v* GE were not mechanically strong, and the 3D structures collapsed during the printing process. By increasing the concentration of the crosslinker from 1–2% *w/v*, the CH–GE–PEG constructs were printed successfully and showed better mechanical properties (Figure 4b,c). At 2% *w/v* GE, the printing required the application of higher pressure due to high viscosity of the bioink (Figure 4c).

To enhance the robustness of the bioprinted structures, sodium alginate, a naturally occurring block copolymer, was used as the first layer (Figure 4d). Alginate has been used for bioprinting applications as its dispensability can be modified by changing the viscosity grade of the polymer (based on mannuronic: guluronic acid ratios) as well as using CaCl_2_ to produce crosslinked hydrogels [52]. However, high levels of Ca^2+^ ions can be cytotoxic. Furthermore, the long-term stability is limited under wound exudate conditions due to exchange of Ca^2+^ ions from the constructs with Na^+^ ions from wound exudate, resulting in slow dissolution of the constructs [54]. Therefore, based on existing research [38,55], sodium alginate (10 mL, 6% *w/v*) gel was partially crosslinked using CaCl_2_ (10 mL, 0.5% *w/v*). Subsequently, the partially crosslinked alginate was printed as the first layer of the 3D construct and further exposed to a higher concentration of CaCl_2_ (1.2% *w/v*) to be fully crosslinked. CH–GE–PEG cell-laden bioink was subsequently printed as the second and third layer on top of the alginate layer. Figure 4e,f show robust 3D printed cell-laden CH–GE constructs with strong mechanical properties and self-supportive structures (the bioprinted construct was able to maintain its structural integrity while being handled). The bioprinted constructs were not thermoreversible, as their structure and form remained the same in the incubator. The results show the successful production of self-supporting CH–GE–PEG cell-laden bioink and alginate bioink constructs by depositing layer-by-layer bioinks through a 400 μm diameter nozzle.

The optimisation of the bioink formulations served the following purposes: (i) to maintain a suitable gel viscosity during extrusion, (ii) to enable the construct to be consolidated after printing and (iii) to allow expansion of a 3D cell network appropriately.

### 3.4. MTT Studies

**Figure 5:**
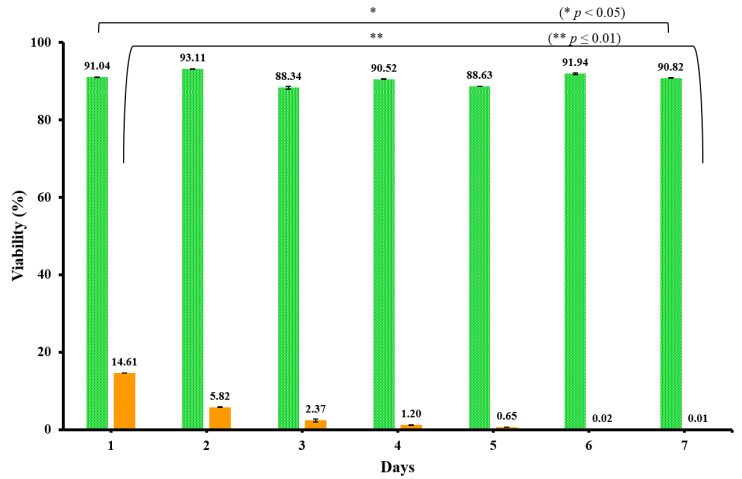
Cytotoxicity (MTT) analysis of the CH–GE–PEG (green) cell-laden 3D constructs with untreated cells as the negative control and Triton-X−100 (orange) as the toxic positive control (*n* = 3, ±SD). Differences between experimental data were considered as statistically significant. * = *p* < 0.05; ** = *p* ≤ 0.01.

MTT was employed to investigate the effect of the CH–GE–PEG bioink as a cell carrier hydrogel on cell viability of HDFs and KCs after printing. This was evaluated daily on separate samples for 7 days, with each sample discarded after MTT analysis. Figure 5 shows the MTT assay profiles for the 3D printed KCs/HDFs within CH–GE–PEG over 7 days. The viability of printed KCs/HDFs (within the bioink) was estimated at 91 ± 2% and 93 ± 1% after 24 and 48 h respectively (Figure 5). Furthermore, the viability of printed KCs/HDFs cells after 3–7 days varied from 88 ± 3% to 93 ± 3%, respectively. According to the ISO guidelines, the cell viability of biocompatible materials should be ≥70% [56]. The MTT assay results indicate that the CH–GE–PEG bioinks are biocompatible, and not cytotoxic, with both HDFs and KCs showing cell viability values greater than 88%. This confirms the biocompatible nature of CH, which is generally regarded as safe and certified for use in various fields including pharmaceutical dosage forms and in skin regeneration following third-degree burns [31].

Pandit and co-workers reported on the interaction between CH and GE, and showed that the resulting constructs possessed higher stiffness, which facilitated the growth and expression of bone cells [57]. Liu and co-authors developed a 3D printing system to fabricate porous CH based scaffolds with the help of pure CH and CH crosslinked with GE [32]. Their results demonstrated that, CH–GE constructs exhibited the highest biocompatibility with osteoblast cells. CH–GE–PEG bioinks showed no statistically significant difference (* represents *p* < 0.05) among the mean of their viability percentage over 7 days. No significant difference between untreated and CH–GE–PEG600 treated was observed while there was significant difference between (CH-GE-PEG and Triton-x). Data are shown as means of six replicates (*n* = 6 ± SD) (DF = 8, F = 1.45301).

Generally, porosity and permeability of the gel plays a key role for cell survival and a porosity measurement would have been ideal. However, the fact that the high cell viability was maintained during 7 days of culture reflects the high likelihood that the bioprinted construct has suitable porosity and permeability for nutrient transportation and waste removal. 

### 3.5. Viability of 3D Printed Constructs Using Live/Dead Staining

**Figure 6:**
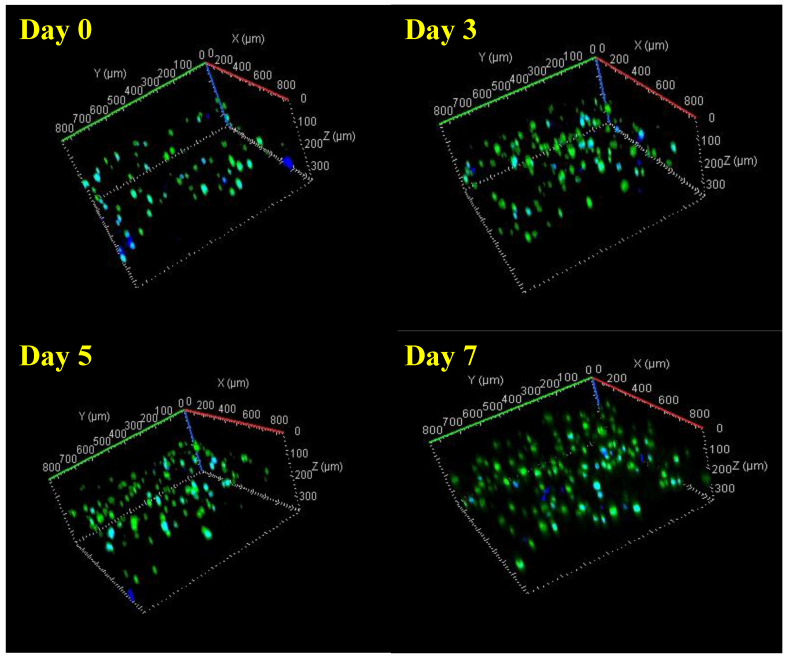
Confocal images of bioprinted CH–GE–PEG cell-laden constructs throughout 7 days showing live (green) and total number of the cells (blue). All images shown in this graph are representative of three independent experiments with similar results.

The most important factor for printing cell-laden CH–GE–PEG constructs is to ensure that the bioink is dispensed with the minimum force, as the shear force applied could cause cell damage, and thereby, reduce cell viability in the printed constructs. Viability of the cells was further investigated to distinguish between live cells (calcein green) and the total number of cells (DAPI blue) for both HDFs and KCs in co-culture. Confocal images showed a high cell viability for all the bioprinted constructs at all time points (Figure 6). This resulted in a final (day 7) viability of 90% (day 0 = 85 ± 1%, day 3 = 87% ± 3, day 5 = 92 ± 2% and day 7 = 90 ± 4%), with the number of cells increasing during this time period.

**Figure 7:**
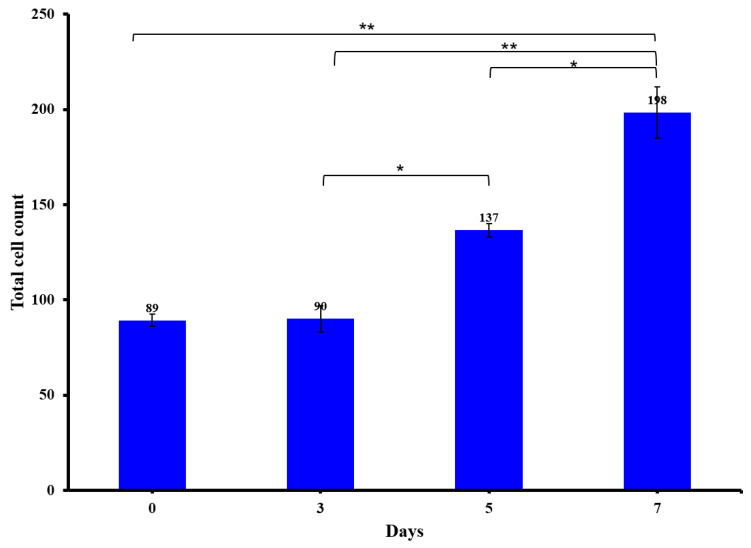
Total cell counts from maximum intensity project of the bioprinted CH–GE–PEG cell-laden constructs throughout seven days (*n* = 3, ±SD). Differences between experimental data at * = *p* < 0.05; ** = *p* ≤ 0.01 were considered as statistically significant.

The number of cells doubled (*n* = 3), from day 0 to day 7, indicating cell survival and proliferation within the hydrogel (Figure 7). Confocal images (Figure 6) showed that a low cytotoxicity generated by the printing process and the overall viability of HDFs/KCs in the printed construct was to 88.5%, immediately after printing. Although cell viability increased with time, the doubling times of these cells in 3D are longer than in 2D [58]. Given that cells are still metabolically active (Figure 5) and cell number is increasing, the increase in doubling time may just be a consequence of the new culture conditions, which is supported within the literature of 3D culture of these cell types [59]. Furthermore, these images are made into maximum intensity projections (MIPs) and the analysis was carried out in Fiji; and these have been included as part of a Supplementary Materials. In Figure 7, where the cell counts have been shown, the error bars are very small (*n* = 3). In terms of other methods, we corroborated these data with MTT, since flow cytometry was not suitable because the cells were not in solution, but rather were within the 3D construct.

It is worth mentioning that images were taken to identify HDFs and KCs separately within the mixture using antibodies specific to the keratinocytes (Pan-Keratin (C11) mouse antibody) to identify individual growth, morphology and spread. However, due to the autofluorescence of the bioink (derived from GE), it was impossible to image [60]. The data demonstrate that the formulated bioink and the parameters employed in this research did not have a negative impact on the embedded cells and promoted cell viability without disrupting the printed samples.

Comparing the MTT and live/dead assays, the doubling time for MTT was 4.392 days, whilst that for the live/dead assay was 1.734 days. These results demonstrate the biocompatibility of the bioink for cell maintenance and viability. However, this structure was not bioactive and did not support cell growth and proliferation as is seen by the MTT and live/dead assay differing in doubling time. This is further supported by the SEM imaging of cell morphology being round and not adopting the typical morphology of fibroblasts being more elongated. Furthermore, cell seeding density may have also been a factor in the observed differences, because the MTT assay was seeded with a total of 1 × 10^4^ cells/mL, whilst the seeding of the gel for the live/dead assay was a total of 1 × 10^6^ cells/mL. However, this will require further investigations in the future.

### 3.6. Scanning Electron Microscopy (SEM) Analysis

**Figure 8:**
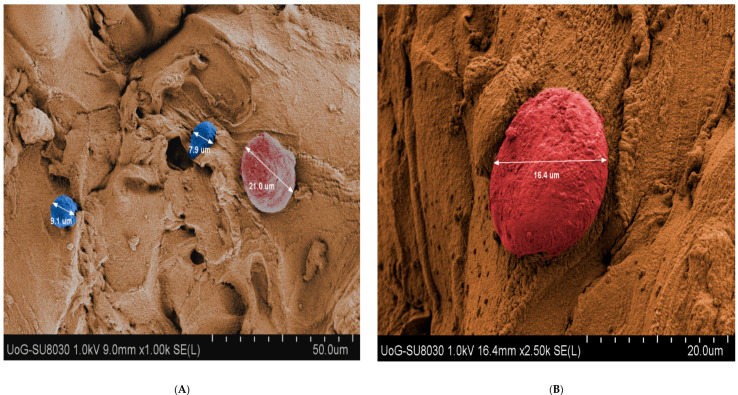
(**A**) SEM image of HDFs (red) and KCs (blue) within 3D bioprinted construct at day 1, (**B**) SEM image of HDFs and KCs within 3D printed construct at day 7 under various magnifications.

KCs and HDFs within CH–GE–PEG constructs were studied using SEM and demonstrated a round shape morphology. As shown in Figure 8, there was a size difference between cells where the larger cells were attributed to the HDFs (red) (20 µm) and the smaller to KCs (blue) (10 µm); nevertheless, this assumption requires further characterisation studies. It is worth mentioning that KCs may form clones if they are 11 µm or less in diameter, but are irreversibly committed to further enlargement and terminal differentiation if they are 12 μm or more in diameter [61] (KCs in SEM images are 9 and 7.9 µm). When a founding cell of 11 µm or less forms a small rapidly growing clone in culture, the cells of that clone can find new colonies even when their diameter is as large as 20 μm.

The images of the scaffolds at low magnification revealed circular shaped macropores ranging in sizes from 2 to 7 µm. Porosity of the materials is essential to support supply of cells deep within the construct with sufficient nutrients during cultivation to maintain their viability. This indicates that the 3D bioprinted construct with the pores, as described above, supports distribution of medium throughout the 3D structure. When KCs and HDFs are grown on tissue culture flasks, the KCs showed a polygonal shape, while HDFs showed a spindle shape [62]. The circular shape of KCs and HDFs suggests that they do not spread their lamellipodium or filopodium, initially (Figure 8A). However, after some time (Figure 8B), these projections can be easily seen. These structures are usually observed in cells undergoing division or migrating, which suggests that the cells are metabolically active and mobile. The ability to adhere is critical to structural organisation and differentiation.

## 4. Conclusions

In this study, we developed novel crosslinked CH–GE bioink formulations laden with KC and HDF cells that were used to fabricate 3D printed constructs for potential skin regeneration after injury. The rheological properties of CH–GE bioinks can be tailored to enhance printability and geometric accuracy. The careful design of bioinks resulted in high cell viability for at least 7 days within the 3D constructs (>85%) for both cell lines due to the low shear stress applied on cells during printing. Our approach overcomes current printing limitations with extrusion-based bioprinting, where existing studies in bioprinting KCs presented low cell viability (40–80%). Furthermore, the developed bioinks facilitated the formation of macropores, which in turn, allowed sufficient transfer of nutrition to maintain viability of the embedded cells, though it did not support proliferation. However, further optimisation is required, including combination with biopolymers naturally present in skin, to support proliferation and additional histological characterisation over a longer time to confirm the formation of full thickness epidermal and dermal skin layers.

## Figures and Tables

**Table 1:** Optimised printing process parameters of designed bioinks following initial development. (CH-chitosan; GE-genipin; PEG-polyethylene glycol).

Bioinks	Pressure (kPa)	Speed (mm/s)	Infill Density (%)
CH–GE–PEG (0.5, 1 and 2% *w/v* GE)	25	4	40
Alginate	10	6	40
CELLINK START^®^	28	5	35

**Table 2:** Rheological properties of crosslinked CH–GE bioinks at various GE concentrations (*n = 3*, ±SD).

Samples	Viscosity (Pa s)			
	25 °C		37 °C	
	0.1 s^−1^ (Shear Rate)	1 s^−1^ (Shear Rate)	0.1 s^−1^ (Shear Rate)	1 s^−1^ (Shear Rate)
CH–PEG	63.4 (±2.0)	11.1 (±1.0)	50.3 (±5.0)	9.1 (±1.0)
CH–GE–PEG (0.5% *w/v* GE)	91.3 (±3.0)	38.2 (±1.0)	80.2 (±3.0)	10.2 (±1.0)
CH–GE–PEG (1% *w/v* GE)	554.2 (±12.0)	73.0 (±2.0)	494.8 (±15.0)	70.8 (±1.0)
CH–GE–PEG (2% *w/v* GE)	743.0 (±14.0)	108.1 (±5.0)	707.8 (±10.0)	98.2 (±3.0)
Commercial bioink	718.5 (±21.0)	19.5 (±3.0)	527.0 (±11.0)	17.4 (±2.0)

## Supplementary Materials

The following are available online at https://www.mdpi.com/1999-4923/12/6/550/s1, Figure S1: Maximum intensity projection images of bioprinted CH–GE–PEG cell-laden constructs throughout seven days.
