# Supplementary material for: Bioprinting and Preliminary Testing of Highly Reproducible Novel Bioink for Potential Skin Regeneration
Source: Pharmaceutics. 2020 Jun 13;12(6):550. doi: 10.3390/pharmaceutics12060550 (PMC7356948; doi:10.3390/pharmaceutics12060550)
Supplement: Supplementary file 1 [file pharmaceutics-12-00550-s001.pdf]

pharmaceutics

MDPI

Supplementary Materials: Bioprinting and Preliminary Testing of Highly Reproducible Novel Bioink for Potential Skin Regeneration

Forough Hafezi, Susan Shorter, Atabak Ghanizadeh Tabriz, Andrew Hurt, Victoria Elmes, Joshua Boateng * and Dennis Douroumis *

Maximum Intensity Projection Images of Bioprinted CH–GE–PEG Cell-Laden Constructs Throughout Seven Days.

Cell counts were calculated based on maximum intensity projection (S1) of acquired Z stacks of the 3D printed bioink. Cell viability and density were evaluated at day 0 (immediately after printing), 3, 5 and 7 (n = 3). Corresponding to figure 7 in the main paper. As can be seen from the images, the cell density does not look too clustered when in 2D. In Figure 7 where the cell counts have been shown, the error bars are very small (n = 3).

Day 0

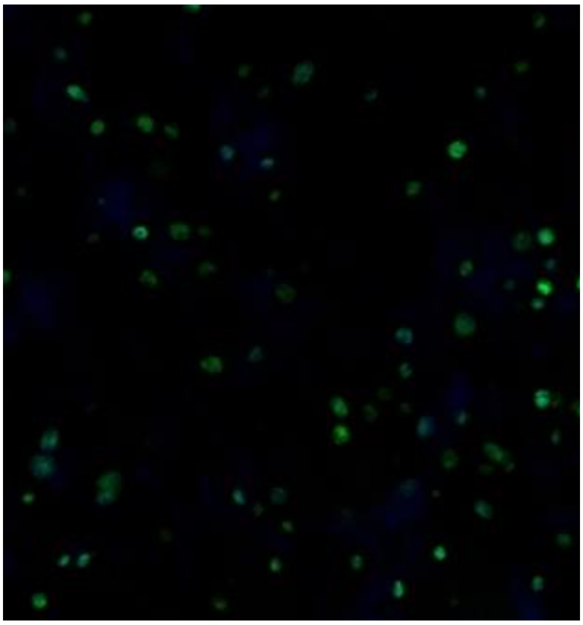

Day 3

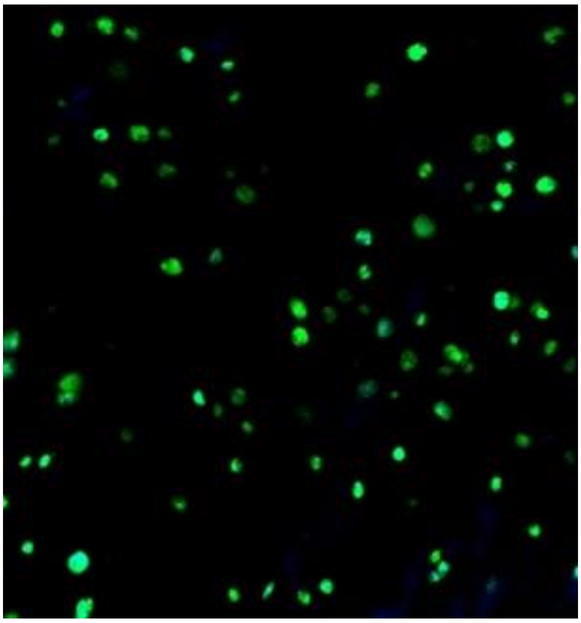

Figure S1. *Cont.*

Day 5 Day 7

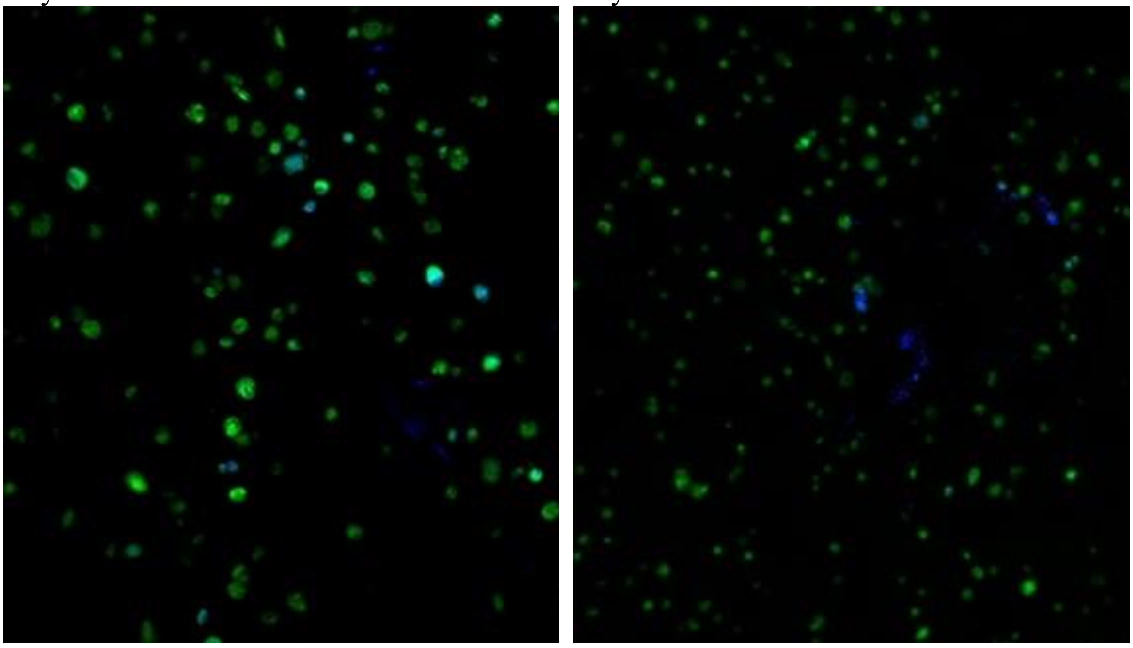

MIP of cells, used for cell counting.

Figure S1. Representative maximum intensity projections of HDFs and KCs printed within a 3D bioink. Stained for calcein AM (green) and DAPI (blue) at day 0 (A)(immediately after printing), day 3(B), day 5(C) and day 7(D). Composite images green and blue labelled cells, mark living cells, whilst blue only denote dead cells.
